# Variability in the Causes and Delay Factors Contributing to Maternal Mortality: Evidence From Maternal Death Surveillance Reports of 22 African Countries

**DOI:** 10.1111/1471-0528.18342

**Published:** 2025-08-28

**Authors:** Francis G. Muriithi, Charles A. Ameh, Ruth W. Gakuo, Caitlin R. Williams, Adam Devall, Arri Coomarasamy, Sue Fawcus

**Affiliations:** ^1^ Department of Metabolism and Systems Science, School of Medical Sciences, College of Medicine and Health University of Birmingham Birmingham UK; ^2^ Department of International Public Health, Emergency Obstetric and Quality of Care Unit Liverpool School of Tropical Medicine Liverpool UK; ^3^ Department of Nursing, Queen's Medical Centre Nottingham University Hospitals NHS Trust, Lenton Nottingham UK; ^4^ UNDP/UNFPA/UNICEF/WHO/World Bank Special Programme of Research, Development and Research Training in Human Reproduction (HRP), Department of Sexual and Reproductive Health and Research, World Health Organization Geneva Switzerland; ^5^ Department of Obstetrics and Gynaecology University of Cape Town Cape Town South Africa

**Keywords:** developing countries: obstetrics and gynaecology, maternal mortality, maternity services, obstetric haemorrhage

## Abstract

**Objective:**

To examine the variability in causes of maternal deaths in African countries using the World Health Organization (WHO) International Classification of Diseases Maternal Mortality (ICD‐MM) framework and the contributing factors using the three delays framework.

**Design:**

Secondary data analysis.

**Setting:**

African countries.

**Sample:**

National maternal death review reports.

**Methods:**

A framework analysis of data extracted from maternal death review reports utilising the ICD‐MM and three delays analytical frameworks.

**Main Outcome Measures:**

Proportions of the causes of maternal death and contributing factors.

**Results:**

Twenty‐two reports published between 2009 and 2022 were included, comprising 18,321 maternal deaths. The mean proportions were: 73% direct, 20% indirect, 8% unspecified, and 1% coincidental. The causes of death consisted of: 6% abortion‐related; 16% hypertensive disorders; 33% obstetric haemorrhage; 9% infection; 10% other direct complications; 3% complications of management; 20% non‐obstetric complications; 8% unknown; and 1% coincidental. All three delays contributed to maternal deaths. The third delay: receiving adequate care, was the most common in all countries except Ethiopia, where the first delay: deciding to seek care, dominated. On average, the first, second, and third delays contributed to 26%, 15%, and 61% of deaths, respectively.

**Conclusion:**

A renewed focus on the quality of care within health facilities, alongside addressing pre‐facility contributing factors, could re‐ignite progress in reducing the burden of preventable maternal deaths.

## 1 Introduction

Maternal mortality in Africa remains alarmingly high; in 2020, the region accounted for 70% of preventable maternal deaths globally, and progress has stagnated in recent years [1, 2]. The consequences of maternal mortality extend beyond the immediate loss of life. They include far‐reaching health, social, and economic consequences for surviving neonates and families at large, often perpetuating cycles of disadvantage and perhaps contributing to the low human development index observed in African countries where the maternal mortality burden is highest [3, 4, 5]. Therefore, conducting maternal death reviews is in the best interest of countries, as they offer critical insights to inform strategies for preventing future maternal deaths and their associated consequences [6].

Maternal death reviews are recommended by the World Health Organization (WHO) [6, 7]. The reviews aim to gain lessons to inform healthcare policy, improve facility‐based care, and strengthen accountability in preventing maternal deaths [8, 9]. Various maternal death review methodologies exist, including Community‐Based Maternal Death Reviews, Facility‐Based Maternal Death Reviews, Confidential Enquiries into Maternal Deaths (CEMD), Maternal Death Surveillance and Response (MDSR), and Maternal and Perinatal Death Surveillance and Response (MPDSR) [10, 11, 12, 13]. While CEMD is considered the most comprehensive approach, many African countries opt for the facility level reviews that is, MPDSR, due to its feasibility and cost‐effectiveness [6, 10, 11, 14].

Countries which opt for facility‐level reviews argue that they allow for timely analysis and integration into governance and risk management processes, ensuring a rapid response to prevent future deaths; however, they are prone to bias in settings with a culture of blame [13, 15]. On the other hand, national reviews, such as CEMD, provide broader insights for system‐wide improvements but may face challenges in implementation due to time and costs involved, generalisability (as they cover the entire country, not a sub‐region or individual facility and its catchment), and impact assessment at sub‐national levels [16, 17]. Beyond the numbers and causes of maternal deaths, maternal death review reports include data on contributing factors: critical insights that are often missing from global maternal mortality estimate reports [11, 18].

A WHO group tracking progress towards the achievement of Sustainable Development Goal target for maternal mortality ratio of below 70 per 100 000 deaths by 2030 published an analysis on global and regional causes of maternal deaths; however, their analysis neither captured nor compared the contributing factors between countries [19]. Such data on factors contributing to maternal deaths are often captured and reported in maternal death review reports.

Published studies drawing on data from maternal death review reports have largely focused on the maternal death review implementation processes and the lessons learnt [10, 11, 17, 20]. One study involving 30 low‐ and middle‐income countries (LMICs) from 2015 to 2019 reported an upward trend in maternal death notification and review, with average annual coverage rates of 28% for notifications and 13% for reviews [10]. A content analysis of 56 review reports from 32 LMICs found considerable variation in report comprehensiveness and format, limiting their comparability and utility [11]. In addition, a review of 13 confidential enquiry systems identified a lack of monitoring of follow‐up actions in response to recommendations [17]. A comparative analysis of country case studies revealed variability in maternal death review implementation highlighting opportunities for learning between countries [20].

A common theme across the above studies is that maternal death review reports contain valuable data; however, they are often government‐owned, difficult to access, and heterogeneous in both content and format, which hinders comparative analysis. In addition, there is no analysis of the patterns of contributing factors to maternal deaths across African countries where maternal mortality is highest. Addressing this knowledge gap could better inform the design of context‐specific interventions and inform policy improvements tailored to diverse health system settings.

This study aims to address the existing knowledge gaps by utilising maternal death review data to examine and describe variability in the causes of maternal deaths using one uniform framework (the WHO ICD‐MM classification framework) and in contributing factors using the Three Delays model [21, 22, 23, 24, 25, 26]. To achieve this, we asked the following research question: What is the pattern of the causes of maternal death, classified using the WHO ICD‐MM framework, and their contributing factors, assessed using the Three Delays model, across African countries?

## 2 Methods

This study draws on data from national maternal death review reports across 22 African countries. The analytical frameworks, procedures for sourcing, preparing, and organising the reports, as well as the methods for data extraction and analysis, are detailed below.

### 2.1 Analytical Frameworks

We utilised two analytical frameworks: (1) International Classification of Diseases Maternal Mortality (ICD‐MM) and (2) Three Delays Framework: first (delay in seeking care), second (delay in reaching care), and third (delay in receiving adequate care) [22, 23, 24, 25, 26]. The ICD‐MM framework aims to standardise the collection, analysis, and interpretation of data on maternal deaths [22, 23, 24]. Under the ICD‐MM framework, maternal death review data are categorised into nine causal groups for cross‐country comparability, namely: Group 1 (Pregnancies with abortive outcome), Group 2 (Hypertensive disorders), Group 3 (Obstetric haemorrhage), Group 4 (Pregnancy related infection), Group 5 (Other direct obstetric complications such as amniotic fluid embolism), Group 6 (Unanticipated complications of management such as bowel injury at caesarean section), Group 7 (Non‐obstetric complications such as medical co‐morbidities), Group 8 (Unknown or undetermined such as a death at home with no witnesses, no medical records and in whom no autopsy was conducted or in whom autopsy findings were inconclusive) and Group 9 (Coincidental causes such as death of a pregnant woman following a road traffic accident) [18, 19, 20, 21]. The factors contributing to the causation of maternal deaths are classified using the Three Delays Framework: First delay (delay in seeking care), Second (delay in reaching care), and Third (delay in receiving adequate care) [25, 26].

### 2.2 Sourcing and Preparation of Maternal Death Review Reports

There is a scarcity of published maternal death review reports [14]. Therefore, FGM used multiple approaches between January 2023 and January 2024 to source for the reports. First, FGM screened the 2016 World Health Organization/United Nations Population Fund (WHO/UNFPA) MSDR baseline survey “Time to Respond” to identify countries that had responded to the global survey on global implementation of MDSR and carried out CEMD, MDSR, MPDSR, MDRs, national maternal or/and perinatal mortality reviews, enabling a targeted search for maternal death review reports [27]. FGM identified 31 countries for a targeted search (see Table 1 in the cited report) [27].

In addition, FGM searched PUBMED and Google (Google LLC, California, USA) and Google Scholar (Google LLC, California, USA) with the search terms: “X” AND (CEMD OR MPDSR OR MDSR OR “MATERNAL DEATH REVIEW” OR “MATERNAL MORTALITY REPORT” OR “MATERNAL DEATHS REPORT”) – Where X is the specific country name for each of the 54 African countries. The first ten pages of each search were examined for suitable reports. The last search was on 26/01/2024 and an update on 6/7/2025. FGM limited the search to the period between 1987 (commencement of the Safe Motherhood Initiative) and 2015. The Search Strategies are presented in File S1.

In the same period, FGM searched the websites of the respective Ministries of Health and contacted technical officers at the WHO Regional Office for Africa by text message requesting any maternal death review reports they might have, the WHO Global MPDSR working group consisting of Technical Officers drawn from the WHO, UNFPA and academic experts of which FGM and CAA were members. All retrieved reports were collated and deduplicated by FGM and stored in an online Dropbox folder (Dropbox Inc., San Francisco, California, USA) in electronic format (available on request to the corresponding author).

### 2.3 Organising the Reports—Inclusion and Exclusion Criteria

Two researchers, FGM and RWG, independently screened the maternal death review reports using pre‐set criteria. Any discrepancies were resolved by consensus in consultation with one of the other co‐investigators. We included national reports written in any language that used CEMD, MDSR, or equivalent maternal death review methods [7, 13, 23]. We excluded sub‐national reports if a national report was available. For countries with multiple reports, only the most recent was included. Collated statistical reports were excluded as they lacked formal maternal death review.

### 2.4 Data Extraction and Analysis

First, reports in non‐English languages were translated by FGM using Google Translate (Google LLC, California, USA), an online multilingual neural machine translation software which has an accuracy of 85%–97% for scientific articles [28, 29]. Due to limited word counts, this translation was done page by page and not the entire report. To validate translations, French and Portuguese native speakers conducted reverse human translations of selected excerpts: a pragmatic methodological approach used in other health research where data were multilingual [30, 31]. The results of the translation validation are reported in File S2.

Second, FGM systematically defined extraction variables by reviewing report structures, identifying common categories, and developing a data extraction proforma. The data extraction proforma was designed based on the WHO International Classification of Diseases Maternal Mortality (ICD‐MM) framework and the three‐delays frameworks [21, 22, 23].

Third, the data extraction proforma was piloted by FGM and RWG on a Google form linked to a Google Sheets spreadsheet.

Finally, two researchers, FGM and RWG, independently extracted and compared the data. Any discrepancies were resolved by consensus in consultation with one of the other co‐investigators. The completed data extraction sheet is presented as File S2.

Following extraction, a descriptive framework analysis was performed using Microsoft Excel software (Microsoft Corporation, Washington, USA). Variability in the causes of maternal death was evaluated by comparing the proportions as percentages across different ICD‐MM groups within and between countries, while variability in contributing factors was assessed by comparing the proportions of the three delays within and between countries. The findings were reported as per the Strengthening the Reporting of Observational Studies in Epidemiology (STROBE) guideline [32].

## 3 Results

Our combined sourcing strategy yielded a total of 341 maternal death review reports. After removal of duplicates, articles and reports from other continents, 80 maternal death review reports from 33 African countries remained. A further 58 were excluded based on our previously described exclusion criteria. Reports from 22 countries were eligible for inclusion into the analysis. We did not identify a single maternal death review report from 21 African countries ‐ amongst them, countries with a high burden of maternal mortality and those facing conflict such as South Sudan and Chad.

**FIGURE 1:**
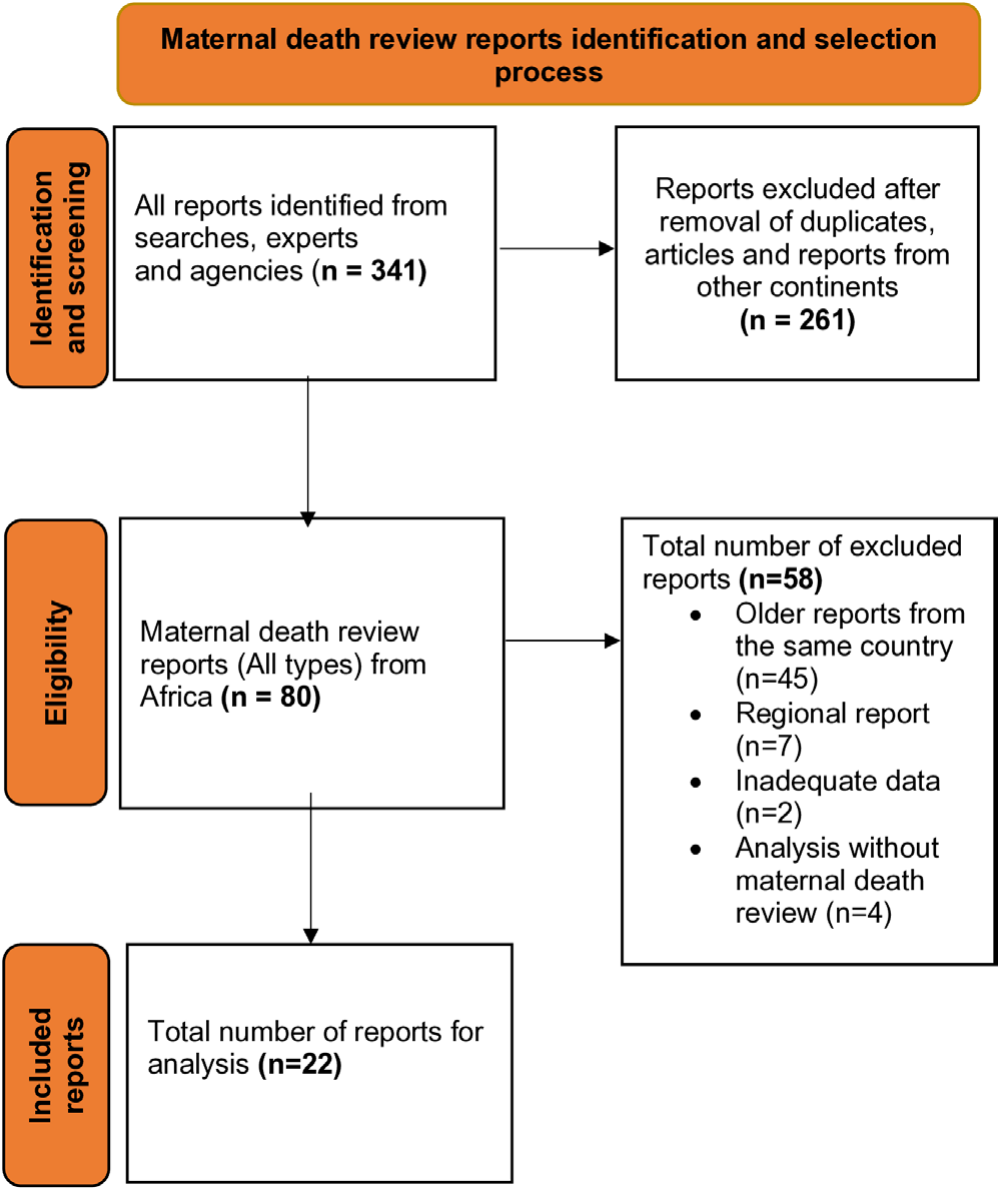
Prisma flow chart illustrates the process of maternal death review reports identification and screening for eligibility.

The identification and selection process of the reports for inclusion is illustrated in Figure 1.

### 3.1 Characteristics of Included Maternal Death Review Reports

Thirteen reports were in English, eight in French, and one in Portuguese. The validated Google Translate translation accuracy was 100% for French and 90% for Portuguese.

The reports were published between 2010 (Lesotho and Morocco) and 2024 (Kenya). Collectively, they reported 18 321 reviewed maternal deaths that occurred between 2009 and 2022. The proportion of reported maternal deaths that were reviewed was variable and ranged from 37% in the Democratic Republic of Congo to 100% in the Republic of Congo, Ethiopia, Côte d'Ivoire, Madagascar, Malawi, Rwanda, Sierra Leone, Tanzania, and Zambia.

The included reports consisted of seven (32%) CEMD, 12 (55%) MPDSR/MDSR, two (9%) maternal death review cross‐sectional studies, and one (5%) national audit report. There were various approaches to conducting the maternal death reviews. The most common approach was the aggregation of subnational (from communities and health facilities, districts or counties, provinces, or regions) maternal death review reports in 11 (50%) reports. This was followed by the CEMD approach in seven (32%) reports. Lastly, national reviews of maternal deaths were in four (18%) reports. Regarding the use of analytical frameworks, nine (41%) reports used the three delays analytical framework, one (5%) report had both the three delays and pathway to survival framework, and the remaining nine (41%) reports analysed the contributing factors based on levels of system failure. Three (14%) reports did not utilise any analytical framework. The data on the characteristics of included maternal death review reports are presented in File S2.

### 3.2 Variability in the Causes of Maternal Deaths by ICD‐MM Groups

Our comparative analysis of the causes of maternal deaths by ICD‐MM groups revealed two main levels of variation. First, there was variation in the magnitude of the contribution of all ICD‐MM groups across countries. Second, there was variation in the leading cause of maternal deaths. In 16 countries (73%), the leading cause of maternal deaths was ICD‐MM group 3, that is, Obstetric haemorrhage. These countries were Benin, Democratic Republic of Congo, Congo, Ethiopia, Côte d'Ivoire, Kenya, Lesotho, Madagascar, Mauritania, Morocco, Mozambique, Rwanda, Sierra Leone, Tanzania, Uganda, and Zambia. In five countries (23%), namely Eswatini, Malawi, Namibia, Niger, and South Africa, ICD‐MM Group 7 deaths, that is, non‐obstetric complications, were the leading causes. In Botswana, ICD‐MM Group 1 deaths, that is, pregnancies with an abortive outcome, were the leading cause of maternal deaths.

Hypertensive disorders (group 2) were the second most frequent cause in 11 countries, namely Botswana, Ethiopia, Kenya, Lesotho, Mauritania, Morocco, Mozambique, Sierra Leone, Tanzania, Uganda, and Zambia.

These findings are presented in Table 1.

**TABLE 1:** Causes of maternal deaths by ICD‐MM groups.

Country/Year of Report	Number of deaths audited	Proportions of the causes of maternal deaths by ICD‐MM groups (G1–G9)								
		G1	G2	G3	G4	G5 direct complications	G6	G7	G8	G9
Benin 2019	380	1%	12%	34%	6%	11%	0%	11%	22%	4%
Botswana 2014	216	24%	20%	20%	8%	6%	2%	19%	1%	0%
Democratic Republic of Congo 2017	91	3%	4%	51%	13%	4%	0%	8%	11%	1%
Congo 2014	223	—	17%	56%	12%	1%	4%	9%	—	—
Eswatini 2016	91	13%	9%	15%	9%	10%	0%	40%	9%	0%
Ethiopia 2011	1010	3%	19%	41%	9%	19%	1%	31%	4%	—
Côte d'Ivoire 2017	2387	4%	9%	42%	4%	2%	—	20%	28%	—
Kenya 2024	1124	5%	23%	38%	12%	—	8%	9%	7%	—
Lesotho 2010	60	8%	25%	32%	3%	—	10%	18%	2%	2%
Madagascar 2011	119	10%	20%	30%	21%	—	—	18%	—	—
Malawi 2014	375	8%	8%	11%	9%	7%	—	30%	5%	—
Mauritania 2019	170	1%	31%	33%	2%	25%	1%	9%	—	—
Morocco 2010	313	—	18%	32%	7%	15%	—	13%	5%	—
Mozambique 2017	816	7%	18%	41%	10%	5%	0%	17%	1%	—
Namibia 2016	80	6%	19%	20%	9%	—	3%	39%	5%	—
Niger 2015	1128	—	18%	25%	12%	19%	—	26%	—	—
Rwanda 2015	294	3%	9%	35%	17%	—	—	19%	17%	—
Sierra Leone 2020	579	3%	17%	43%	9%	13%	—	10%	4%	—
South Africa 2019	3655	5%	15%	16%	5%	6%	3%	43%	7%	—
Tanzania 2018	1744	3%	19%	38%	8%	8%	3%	11%	1%	—
Uganda 2022	1096	5%	14%	41%	9%	12%	2%	12%	5%	—
Zambia 2018	2370	8%	13%	39%	7%	2%	1%	22%	7%	1%
Total/Mean	18 321	6%	16%	33%	9%	10%	3%	20%	8%	1%

*Note:* A dash (—) means no data were available for that group. The cell with the greatest proportion in a country is shaded red. ICD‐MM Groups: G1 = Pregnancies with abortive outcome, G2 = Hypertensive disorders in pregnancy, childbirth, and the puerperium, G3 = Obstetric haemorrhage, G4 = Pregnancy related infection, G5 = Other obstetric complications, G6 = Unanticipated complications of management, G7 = Non‐obstetric complications, G8 = Unknown/undetermined and G9 = Coincidental causes.

### 3.3 Variability in the Causes of Maternal Deaths: A Comparison of Direct, Indirect, Unspecified, and Co‐Incidental Deaths

We found that when maternal deaths are grouped into direct, indirect, unspecified, and coincidental categories, the most common leading category in all countries was direct maternal deaths. However, the magnitude varied from one country to another: the range in the direct category varied from 43% (Malawi, 2014) to 92% (Mauritania, 2019). The indirect category ranged between 8% (Democratic Republic of Congo, 2017) and 43% (South Africa, 2019). The unspecified category ranged from 1% (Botswana, 2014; Tanzania, 2018) to 28% (Côte d'Ivoire, 2017), while the coincidental category ranged from 0% (Botswana, 2017; Eswatini, 2016) to 4% (Benin, 2019). These findings are presented in Table 2.

**TABLE 2:** Causes of maternal deaths by direct, indirect, unspecified, and co‐incidental groups.

Country/Year of report	Number of deaths audited	Total direct	Indirect	Unspecified	Co‐incidental
Benin 2019	380	64%	11%	22%	4%
Botswana 2014	216	81%	19%	1%	0%
Democratic Republic of Congo 2017	91	76%	8%	11%	1%
Congo 2014	223	91%	9%	—	—
Eswatini 2016	91	56%	40%	9%	0%
Ethiopia 2011	1010	83%	31%	4%	—
Côte d'Ivoire 2017	2387	60%	20%	28%	—
Kenya 2024	1124	86%	9%	7%	—
Lesotho 2010	60	78%	18%	2%	2%
Madagascar 2011	119	82%	18%	—	—
Malawi 2014	375	43%	30%	5%	—
Mauritania 2019	170	92%	9%	—	—
Morocco 2010	313	71%	13%	5%	—
Mozambique 2017	816	81%	17%	1%	—
Namibia 2016	80	56%	39%	5%	—
Niger 2015	1128	74%	26%	—	—
Rwanda 2015	294	64%	19%	17%	—
Sierra Leone 2020	579	86%	10%	4%	—
South Africa 2019	3655	50%	43%	7%	—
Tanzania 2018	1744	79%	11%	1%	—
Uganda 2022	1096	83%	12%	5%	—
Zambia 2018	2370	70%	22%	7%	1%
Total/Mean	18 321	73%	20%	8%	1%

*Note:* A dash (—) means no data were available for that group. The cell with the greatest proportion in a country is shaded red.

### 3.4 Variability in the Factors Contributing to Maternal Deaths by the Three Delays Framework

Data on the contribution of the three delays to the causes of maternal deaths were available from 17 of 22 countries. In 16 (94%) of those countries, the third delay (delay in receiving adequate care) was the most common. In Ethiopia (2011), the first delay was the most common. These findings are presented in Table 3.

**TABLE 3:** Comparison of the proportions in the factors contributing to maternal deaths by the 3‐delays framework.

Country/Year of report	Number of maternal deaths audited	1st delay	2nd delay	3rd delay
Benin 2019	380	5%	16%	60%
Botswana 2014	216	33%	13%	53%
Democratic Republic of Congo 2017	91	30%	6%	64%
Congo 2014	223	7%	10%	83%
Eswatini 2016	91	21%	3%	100%
Ethiopia 2011	1010	67%	38%	49%
Côte d'Ivoire 2017	2387	—	—	—
Kenya 2024	1124	40%	19%	41%
Lesotho 2010	60	9%	0%	91%
Madagascar 2011	119	16%	16%	68%
Malawi 2014	375	35%	12%	53%
Mauritania 2019	170	—	—	—
Morocco 2010	313	19%	17%	56%
Mozambique 2017	816	9%	14%	77%
Namibia 2016	80	21%	14%	65%
Niger 2015	1128	—	—	—
Rwanda 2015	294	41%	15%	44%
Sierra Leone 2020	579	35%	29%	36%
South Africa 2019	3655	42%	6%	53%
Tanzania 2018	1744	—	—	—
Uganda 2022	1096	20%	34%	45%
Zambia 2018	2370	—	—	—
Total/Mean	18 321	26%	15%	61%

*Note:* A dash (—) means no data were available for that category. The cell with the greatest proportion in a country is shaded red.

## 4 Discussion

### 4.1 Main Findings

We highlight our findings during the conduct of the study as well as in line with our outcome measures. In summary, our main findings were: evidence of variability in the methodology, reporting, and accessibility of maternal death review reports between and within countries; variability in the leading causes of maternal deaths by ICD‐MM category and magnitude; and the delay in receiving adequate care (third delay) as the predominant contributing factor. Each of these findings is discussed further in turn.

Regarding the accessibility of maternal death review reports, we found that not all reports were accessible, perhaps due to government embargoes or concerns about political sensitivity. In some cases, only outdated reports were available, raising concerns about contemporaneous data‐driven decision‐making. In addition, the reports we accessed were variable in format between and within countries, making comparative analysis difficult. Limited accessibility of maternal death review reports has been reported by other scholars [18, 32, 33, 34].

On the variability in the causes of maternal deaths, we found that direct maternal deaths remain the leading causes of audited maternal deaths but with variable magnitude between countries. Obstetric haemorrhage (ICD‐MM Group 3) was the leading cause of maternal deaths. For a few others, it was non‐obstetric complications, pregnancy with abortive outcomes, and hypertensive disorders that were the leading causes. For all, variability in the magnitude of each cause between countries was evident. This finding is important as individual countries adapt global recommendations for tackling the causes of maternal deaths. For example, while the global agenda may be to tackle obstetric haemorrhage, the priority for a country may differ depending on its own distribution in the causes of maternal deaths.

### 4.2 Strengths and Limitations

The main strength of our study is in the novel use of routine maternal death review reports as a source of data and the systematic effort to acquire as many reports as possible to ensure comprehensiveness.

Despite our best efforts, we faced some limitations in accessing the reports. First, we could not find a single open‐access global repository for maternal mortality review reports. While some reports were freely accessible, others were under government embargo and required approval to access them. It is possible that there were more reports which could not be accessed. Therefore, the scope of our analysis was limited to the available reports.

A second limitation emanates from the heterogeneity in maternal death review methodology, report formats, and variable timelines of when maternal deaths occurred, when they were reviewed, and when the reports were released. Our approach of combining and comparing maternal deaths that occurred over different years in our study limits the internal validity. Ideally, all included deaths should have been from the same period and with homogeneity in their maternal death review approaches and reporting formats.

Other scholars have reported similar observations of heterogeneity in maternal death review approaches and reporting, suggesting that methodological flaws are fewer when expert panels conduct maternal death reviews and ascribe the cause of maternal death [11, 35]. Therefore, there is an urgent need to recommend standardisation in methodology, reporting, and access to maternal death review reports [23]. The potential benefit of standardisation in methodology and reporting of maternal death reviews is to facilitate the examination of trends and comparisons, facilitating learning from maternal deaths between and within countries [11].

Third, the reports we identified and included were not based on the analysis and reporting of individual maternal deaths, as is the case with coroner's reports [36]. Lack of access to individual maternal death review reports hindered further analysis, verification, and insight beyond what was presented in the reports, particularly details of the third delay problems.

Finally, the use of the Google translation software to translate some reports into English could have led to some inaccuracies arising from the innate limitations of the Google Translate software. We mitigated against this by use of native speakers of French and Portuguese to cross‐check and validate the translations. There could be other more robust AI‐based translation tools which we did not explore.

### 4.3 Interpretation

In comparison with recent literature, a WHO multi‐country analysis reported obstetric haemorrhage as the leading cause of maternal deaths between 2009 and 2020 [19]. This analysis, however, was based on Sustainable Development Goals (SDG) regions and worldwide, not individual countries. It also excluded Human Immunodeficiency Virus/Acquired Immunodeficiency Syndrome (HIV/AIDS) related maternal deaths, which increased the risk of direct maternal mortality by over 5 times in Africa with the risk persisting up to one year postpartum [37, 38]. The reports included in our analysis considered maternal deaths from all categories including indirect causes due to HIV/AIDS ‐ an important problem in African countries.

Published literature links obstetric haemorrhage and related deaths to delays in detection and treatment and substandard medications, as reported in studies from Kenya, Nigeria, South Africa, and Tanzania [39, 40, 41]. Additionally, country‐specific policies and practices may explain variations in leading causes of maternal deaths. For example, South Africa's efforts to reduce obstetric haemorrhage have been successful, therefore highlighting hypertensive disorders as the next leading cause of direct maternal deaths [42]. In South Africa, Zambia, and Eswatini, ICD‐MM Group 7 was the leading cause of indirect maternal deaths, likely due to the high prevalence of HIV and its complications during pregnancy and childbirth [43].

Finally, variations in ICD‐MM Group 1 death may reflect a country's abortion laws, influencing care‐seeking behaviour, access to care, and reporting of abortion‐related maternal deaths [44]. Indeed, abortion‐related maternal deaths are higher in countries with restrictive laws, while legalising abortion can reduce these deaths by 30%–40% [44, 45].

Our findings highlight the need for countries to analyse maternal death patterns to tailor global recommendations to their context [7, 20, 46]. For example, global reports and interventions may target obstetric haemorrhage, but our findings show it is not always the leading cause. Countries should analyse regional and facility‐level variations to advocate for resources and prioritise evidence‐based interventions [47]. Subnational analysis may reveal specific patterns that can prompt in‐depth reviews and guide interventions to tackle individual, health facility, and wider health system factors [48, 49, 50].

Regarding the variability in the contributing factors, our finding regarding the third delay being the leading contributor to maternal deaths in African countries is consistent with the findings of a review paper that examined the contribution of the three delays to the causation of maternal deaths in South‐East Asia, Europe and Africa [51].

Our findings suggest that greater efforts should be directed towards addressing the causes of maternal deaths within health facilities; therefore, tackling the third delay. That said, the third delay rarely contributes to maternal death in isolation; it often occurs alongside the first and second delays, whose adverse effects may cascade into the third, with their cumulative impact resulting in a preventable death [52]. Furthermore, recent studies in Nigerian cities revealed further insights into the difficulties in measuring and accounting for time in the second delay [53, 54]. Therefore, this complex interplay between the delays should be considered while designing and implementing solutions for tackling the causes of preventable maternal deaths.

### 4.4 Recommendations for Practice, Policy, and Research

Based on our study findings, we make the following recommendations:

1. A need for standardisation of approaches to maternal death reviews between countries and the development and adoption of a standard reporting template globally to enhance comparability and facilitate shared learning within and between countries.
2. Governments should not restrict the release of maternal death review reports. Maternal death review reports could be disseminated via the respective Ministry of Health websites. Immediate release and feedback could fast‐track implementation of maternal death review findings to mitigate against the re‐occurrence of similar preventable maternal deaths.
3. Individual countries should consider variations in the causes and factors contributing to maternal deaths in their own setting at subnational levels or by level of care, leading to targeted resource allocation and implementation of clinical and health system interventions.
4. Further research is required to examine trends, explore the potential utility of lessons gained from variability at sub‐national levels and by level of care in informing implementation and intervention decisions at the various levels of the healthcare system.

## 5 Conclusion

There is variability in the methodology, reporting, and accessibility of maternal death review reports, which hinders effective comparisons between countries. We recommend standardisation of methodology and reporting as well as easing accessibility to reports via an open‐access national for example, via a Ministry of Health website or a global repository housed by any of the agencies leading on tackling the burden of preventable maternal deaths for example, WHO, UNFPA, World Bank or global association such as International Federation of Gynaecology and Obstetrics (FIGO).

There is evidence of country‐level variability in the leading causes of maternal deaths. We recommend that individual countries should examine variability in their maternal death burden to ensure that relevant evidence‐based clinical and health system interventions are implemented. Although all three delays contribute to the causation of preventable maternal deaths, the delay in receiving adequate care (third delay) while women are already within the health facilities is a leading contributor. Greater efforts should be directed towards improving the quality of care given to women within health facilities alongside addressing the other pre‐facility contributing factors.

## Author Contributions

**Francis G. Muriithi:** conceptualisation, methodology, sourcing of reports, data curation, formal analysis, visualisation, writing the first draft of the manuscript, writing – review and editing, project administration. **Charles A. Ameh:** supervision, methodology, sourcing of reports, data curation, writing – review and editing, visualisation. **Ruth W. Gakuo:** data curation, writing – review and editing. **Caitlin R. Williams:** supervision, methodology, writing – review and editing, visualisation. **Adam Devall:** supervision, writing – review and editing. **Arri Coomarasamy:** supervision, funding acquisition, resources, writing – review and editing. **Sue Fawcus:** supervision, methodology, writing – review and editing.

## Ethics Statement

Ethical approval was not sought as this study was based on existing reports, and no individual participant data were collected from patients, families, the public or healthcare providers.

## Consent

The authors have nothing to report.

## Conflicts of Interest

The authors declare no conflicts of interest.

## Supporting information


**File S1:** Search strategies and search output.

**File S2:** Completed data extraction sheet.

## Data Availability

The data that support the findings of this study are available from the corresponding author upon reasonable request.
