# Supplementary material for: Variability in the Causes and Delay Factors Contributing to Maternal Mortality: Evidence From Maternal Death Surveillance Reports of 22 African Countries
Source: BJOG. 2025 Aug 28;133(1):106–15. doi: 10.1111/1471-0528.18342 (PMC12676202; doi:10.1111/1471-0528.18342)
Supplement: Supplementary file 1 — File S1: Search strategies and search output. [file BJO-133-106-s002.docx]

**S1 Search Strategies**

**PUBMED**

“Algeria” OR “Angola” OR “Benin” OR “Botswana” OR “Burkina Faso” OR “Burundi” OR “Cabo Verde” OR “Cape Verde” OR “Cameroon” OR “Central African Republic” OR “Chad” OR “Comoros” OR “Democratic Republic of the Congo” OR “Republic of the Congo” OR “Djibouti” OR “Egypt” OR “Equatorial Guinea” OR “Eritrea” OR “Eswatini” OR “Swaziland” OR “Ethiopia” OR “Gabon” OR “ The Gambia” OR “Ghana” OR “Guinea” OR “Guinea-Bissau” OR “Ivory Coast” OR “Côte d'Ivoire” OR “Kenya” OR “Lesotho” OR “Liberia” OR “Libya” OR “Madagascar” OR” Malawi” OR “Mali” OR “Mauritania” OR “Mauritius” OR “Morocco” OR “Mozambique” OR “Namibia” OR “Niger” OR “Nigeria” OR “Rwanda” OR “Sao Tome and Principe” OR “Senegal” OR “Seychelles” OR “Sierra Leone” OR “Somalia” OR “South Africa” OR “South Sudan” OR “Sudan” OR “Tanzania” OR “Togo” OR “Tunisia” OR “Uganda” OR “Zambia” OR “Zimbabwe”

(("Maternal death"[All Fields] OR "maternal mortality"[All Fields]) AND ("review*"[All Fields] OR "Surveillance and Response"[All Fields] OR "Confidential Enquiry"[All Fields]) AND ("reportable"[All Fields] OR "reporting"[All Fields] OR "reportings"[All Fields] OR "research report"[MeSH Terms] OR ("research"[All Fields] AND "report"[All Fields]) OR "research report"[All Fields] OR "report"[All Fields] OR "reported"[All Fields] OR "reports"[All Fields]) AND ("Algeria"[All Fields] OR "Angola"[All Fields] OR "Benin"[All Fields] OR "Botswana"[All Fields] OR "Burkina Faso"[All Fields] OR "Burundi"[All Fields] OR "Cabo Verde"[All Fields] OR "Cape Verde"[All Fields] OR "Cameroon"[All Fields] OR "Central African Republic"[All Fields] OR "Chad"[All Fields] OR "Comoros"[All Fields] OR "Democratic Republic of the Congo"[All Fields] OR "Republic of the Congo"[All Fields] OR "Djibouti"[All Fields] OR "Egypt"[All Fields] OR "Equatorial Guinea"[All Fields] OR "Eritrea"[All Fields] OR "Eswatini"[All Fields] OR "Swaziland"[All Fields] OR "Ethiopia"[All Fields] OR "Gabon"[All Fields] OR "The Gambia"[All Fields] OR "Ghana"[All Fields] OR "Guinea"[All Fields] OR "Guinea-Bissau"[All Fields] OR "Ivory Coast"[All Fields] OR "Cote d'Ivoire"[All Fields] OR "Kenya"[All Fields] OR "Lesotho"[All Fields] OR "Liberia"[All Fields] OR "Libya"[All Fields] OR "Madagascar"[All Fields] OR "Malawi"[All Fields] OR "Mali"[All Fields] OR "Mauritania"[All Fields] OR "Mauritius"[All Fields] OR "Morocco"[All Fields] OR "Mozambique"[All Fields] OR "Namibia"[All Fields] OR "Niger"[All Fields] OR "Nigeria"[All Fields] OR "Rwanda"[All Fields] OR "Sao Tome and Principe"[All Fields] OR "Senegal"[All Fields] OR "Seychelles"[All Fields] OR "Sierra Leone"[All Fields] OR "Somalia"[All Fields] OR "South Africa"[All Fields] OR "South Sudan"[All Fields] OR "Sudan"[All Fields] OR "Tanzania"[All Fields] OR "Togo"[All Fields] OR "Tunisia"[All Fields] OR "Uganda"[All Fields] OR "Zambia"[All Fields] OR "Zimbabwe"[All Fields])) AND (1987:2025[pdat])

Yield = 512

Eligible 1 – Namibia,
<https://pmc.ncbi.nlm.nih.gov/articles/PMC10618300/pdf/10995_2023_Article_3771.pdf>

Ethiopia
<https://pubmed.ncbi.nlm.nih.gov/34158750/>

Liberia
<https://pubmed.ncbi.nlm.nih.gov/38560688/>

Benin, Nigeria
<https://pubmed.ncbi.nlm.nih.gov/31856173/>

Rwanda
<https://pubmed.ncbi.nlm.nih.gov/26801466/>

Ghana
<https://pubmed.ncbi.nlm.nih.gov/26908958/>

DRC
<https://pmc.ncbi.nlm.nih.gov/articles/PMC11340148/>

Kenya

<https://pubmed.ncbi.nlm.nih.gov/25396027/>

Ondo State, Nigeria

<https://pubmed.ncbi.nlm.nih.gov/31864320/>

Lagos, Nigeria

<https://pubmed.ncbi.nlm.nih.gov/29240754/>

Burkina Faso

<https://pubmed.ncbi.nlm.nih.gov/30647992/>

Malawi

<https://pubmed.ncbi.nlm.nih.gov/37584911/>

Tanzania

<https://pubmed.ncbi.nlm.nih.gov/26670664/>

<https://pmc.ncbi.nlm.nih.gov/articles/PMC4681083/>

Sierra leone

<https://pubmed.ncbi.nlm.nih.gov/38216175/>

Egypt
<https://pubmed.ncbi.nlm.nih.gov/17578356/>

**GOOGLE SEARCH**

("Maternal death “OR "maternal mortality") AND ("review*" OR "Surveillance and Response" OR "Confidential Enquiry") AND "report" AND ("Algeria" OR "Angola" OR "Benin" OR "Botswana" OR "Burkina Faso" OR "Burundi" OR "Cabo Verde” OR "Cape Verde" OR "Cameroon" OR "Central African Republic “OR "Chad" OR "Comoros" OR "Democratic Republic of the Congo" OR "Republic of the Congo" OR "Djibouti" OR "Egypt" OR "Equatorial Guinea" OR "Eritrea" OR "Eswatini" OR "Swaziland" OR "Ethiopia" OR "Gabon" OR "The Gambia" OR "Ghana" OR "Guinea" OR "Guinea-Bissau" OR "Ivory Coast" OR "Cote d'Ivoire" OR "Kenya" OR "Lesotho" OR "Liberia" OR "Libya" OR "Madagascar" OR "Malawi" OR "Mali" OR "Mauritania" OR "Mauritius" OR "Morocco" OR "Mozambique" OR "Namibia" OR "Niger" OR "Nigeria" OR "Rwanda" OR "Sao Tome and Principe" OR "Senegal" OR "Seychelles" OR "Sierra Leone" OR "Somalia" OR "South Africa" OR "South Sudan" OR "Sudan" OR "Tanzania" OR "Togo" OR "Tunisia" OR "Uganda" OR "Zambia" OR "Zimbabwe")

**First 10 pages reviewed**

Cameroon
[**https://www.humanitarianlibrary.org/sites/default/files/2021/08/maternal-mortality-in-cameroon-a-critical-review-of-its-determinants.pdf**](https://www.humanitarianlibrary.org/sites/default/files/2021/08/maternal-mortality-in-cameroon-a-critical-review-of-its-determinants.pdf)

DRC
<https://bmcpregnancychildbirth.biomedcentral.com/articles/10.1186/s12884-022-04783-z>

Namibia
<https://link.springer.com/content/pdf/10.1007/s10995-023-03771-9.pdf>
